# Survey of Gastrointestinal Parasites and Lungworms in Cats and Dogs from Terceira and São Miguel Islands, Azores

**DOI:** 10.3390/pathogens13080648

**Published:** 2024-07-31

**Authors:** Romana Teixeira, Isilda Flor, Telmo Nunes, Carlos Pinto, Maria Constança Pomba, Luís Madeira de Carvalho

**Affiliations:** 1 Centre for Interdisciplinary Research in Animal Health, Faculty of Veterinary Medicine, Lisbon University, (CIISA-FMV-ULisboa), Avenida da Universidade Técnica, 1300-477 Lisboa, Portugal; pinanunes@gmail.com (T.N.); cpomba@fmv.ulisboa.pt (M.C.P.); 2 Associate Laboratory for Animal and Veterinary Sciences (AL4AnimalS), 1300-477 Lisbon, Portugal; 3 Laboratory of Parasitology, Regional Veterinary Laboratory, Vinha Brava, 9700-236 Angra do Heroísmo, Portugal; isildaflor@gmail.com; 4 Faculty of Agricultural and Environmental Sciences, University of the Azores, 9700-042 Angra do Heroísmo, Portugal; carlos.a.pinto@uac.pt

**Keywords:** Azores, dog, cat, parasites, gastrointestinal, lungworms

## Abstract

Parasitic diseases can affect animal health and welfare, and they may also constitute a danger to public health, particularly in island ecosystems. Fecal samples were collected from 205 dogs and 115 cats on the islands of São Miguel and Terceira, Azores archipelago (Portugal), using the Willis flotation technique and modified Baermann method, for further analysis. The overall prevalence of gastrointestinal parasitism in dogs was 53%, with the following results: Ancylostomatidae (hookworms) (42.44%), *Trichuris vulpis* (17.56%), *Toxocara canis* (12.68%) and *Cystoisospora* spp. (4.39%). In cats, the overall prevalence was also 53%, with the following results: *Toxocara cati* (31.3%), Ancylostomatidae (30.43%), *Cystoisospora* spp. (14.78%) and *Trichuris* sp. (0.87%). The prevalence of lungworms was 0.49% in canines and 20.87% in felines, with *Angiostrongylus vasorum* and *Aelurostrongylus abstrusus* species being detected in dogs and cats, respectively. The present survey detected a high prevalence of gastrointestinal infection, in both dogs and cats, probably because the samples came mainly from kennels and catteries and due to the peculiar climatic conditions in this insular territory, with mild temperature and high relative humidity. A considerable prevalence of aelurostrongylosis was also detected (20.87%), so it should be included in the list of differential diagnoses of diseases concerning the respiratory tract in cats of the archipelago.

## 1. Introduction

Parasites are still one of the main problems that strongly affect our pets. Despite being undervalued, parasitic diseases can seriously affect animal health and well-being, and some of them are zoonotic, which in itself constitutes a danger to public health [1,2].

However, the fact that animals often do not show signs of infection leads to the incorrect prophylaxis and treatment of these infections [3].

Also, at veterinary clinics, the administration of antiparasitic drugs is mainly intended for prophylactic purposes or as a first line in preparing a differential diagnosis, so identification and registration are often undervalued or ruled out. This is due to the small number of parasitological records existing at European, national and island autonomous regions. The only study carried out on the island of São Miguel by Afonso-Roque (1995) of terrestrial vertebrates referred only to the existence of certain helminths in the domestic dog (*Ancylostoma caninum*, *Toxocara canis*, *Trichuris vulpis* and *Uncinaria stenocephala*), with no record of their prevalence on the island or region [4]. For this reason, the present study focused on the Azores archipelago, more specifically the two most representative islands of this insular region—Terceira and São Miguel. Dogs and cats were introduced to these islands when the archipelago was first populated by the Portuguese people, in the years 1439 to 1444 [5,6]. Since then, these species have developed a closer relationship with humans, moving from working animals to companion animals. However, these animals also contribute to the loss of biodiversity in ecosystems due to their predatory habits, habitat invasion and spread of infectious agents [7,8].

The dog population on the Azores islands is mainly composed of privately owned animals with free-roaming lifestyles. This scenario is visible not only in rural areas but also in cities. Furthermore, most of the islands have kennels for stray and abandoned animals, where prophylaxis and treatment protocols are not regularly applied. A similar case is observed in the cat population of the Azores, where its impact on biodiversity is due to the numerous cats kept by humans in a state of semi-dependence, especially in rural areas, where the cats have greater contact with the native fauna that constitutes their prey [7,8,9].

Another factor to consider is that the number of dogs and cats traveling to and from this insular territory is increasing in proportion to tourism, which has increased in recent years and represents a risk since these pets can acquire or introduce pathogens globally [10,11].

For this purpose, and to obtain as much information as possible, coprological examinations were carried out on both cats and dogs for a further investigation of gastrointestinal and pulmonary parasites. Moreover, a questionnaire was applied to caregivers to determine risk factors associated with the presence of parasites.

## 2. Materials and Methods

### 2.1. Study Area

**Figure 1:**
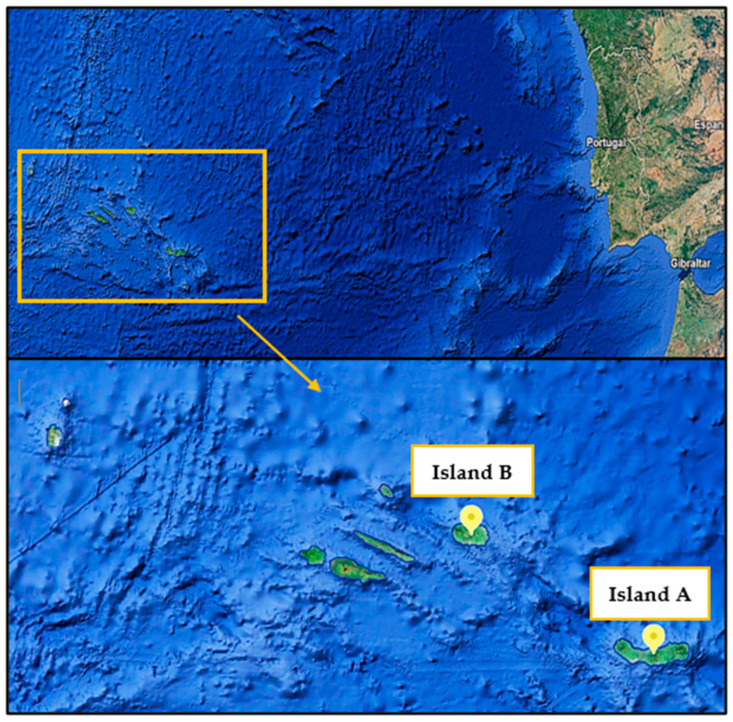
Islands of the Azores Archipelago covered by the study of gastrointestinal parasites and lungworms in cats and dogs living in São Miguel (**Island A**) and Terceira (**Island B**). Available at Google Maps: https://earth.google.com/web/search/S%c3%a3o+Miguel+Island/@37.82350696,-26.49535856,566.44337324a,2282754.15127426d,35y,0h,0t,0r/data=CigJgokCSfh6U9lJTNAESXh6U9lJTPAGWq1TRxVrj5AIRWOmV0hVlHAOgMKATA (accessed on 10 June 2024).

**Figure 2:**
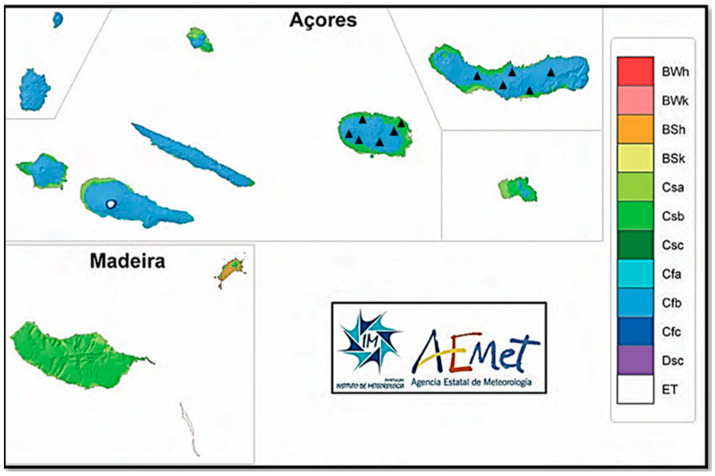
Köppen–Geiger climate classification in the archipelagos of the Azores and Madeira Islands: BWh—hot desert; BWk—cold desert; BSh—hot steppe; BSk—Cold steppe; Csa—temperate with hot and dry summers; Csb—temperate with dry and warm summers; Csc—temperate with dry and cold summers; Cfa—temperate with no dry season and hot summers; Cfb—temperate with no dry season and mild summers; Cfc—temperate with no dry season and cold summers; Dsc—dry summers, subarctic climate; ET—polar tundra [12].

The study took place on two islands of the Azores archipelago, located in the Atlantic Ocean, between September 2019 and January 2020, where a total of 320 samples were collected: 153 on Island A—São Miguel (37.7804° N, 25.4970° W); 167 on Island B—Terceira (38.4315° N, 27.1313° W) (Figure 1). The majority of samples were collected on Terceira Island, given its proximity to the laboratory where the analysis took place. Geographic location is a strong determinant of the climate present on the islands of the archipelago. Therefore, the different islands have different climatic features. Also, within each island, there are climatic asymmetries related to the morphology, geological structure, vegetation and, in some cases, the influence of neighboring islands. Some locations on the islands are recognized for having a well-defined microclimate. Nonetheless, the sample collection took place almost entirely in Cfb locations (Figure 2) distinctive of the general climate classification of the Azores archipelago [12]. Likewise, the level of precipitation differs across the various islands, being higher on islands in the western group and lower on those in the eastern group. Even so, in general, the climate of the Azores is characterized by high levels of air humidity, mild temperatures, low insolation rates, regular and abundant rainfall, and severe winds. The climate is temperate, with average temperatures of 13 °C in winter and 24 °C in summer [13].

### 2.2. Sample Collection

For each fecal sample, a questionnaire was filled out not only to determine predisposing factors but also to record the data of each animal. The evaluated factors were age, sex, breed, lifestyle, exterior access, cohabitation, deworming frequency, diagnosed diseases, origin of samples and length of time spent in shelters. Both species were distributed in two age categories: young (age ≤ 1 year) and adults (age > 1 year).

This questionnaire was preceded by a small pilot survey for which five animal tutors were invited to answer the questions so that the type of questions, extent and time to be answered was reviewed and corrected. The fecal collection was carried out directly from the substrate or sandbox after the animals defecated and then placed in individual plastic bags, identified and stored in a refrigerated isothermal box. The study involved animals from kennels, catteries and tutors, mostly from kennels and catteries, given the high population density present, the greater proximity between the animals and the low deworming frequency. The collected samples were transported to the Regional Veterinary Laboratory (LRV) on Terceira Island for macroscopic and microscopic examinations.

The minimum sample size was calculated to perform this pilot study. However, it is important to highlight that there are no records of the number of animals in the dog and cat populations of the Azores archipelago. Therefore, we accessed the epidemiological platform WinEpi 2.0, which allowed us to determine the sample size without knowing the population estimated numbers. For dogs, an expected proportion of 20% was used, which was obtained from another study carried out on Madeira Island, Portugal [14], and a confidence level of 95%. For cats, only the expected proportion differed, using a value of 23%, which was obtained from a study carried out on Gran Canaria Island, Spain [15] (http://www.winepi.net/f101.php, accessed on 4 September 2019 [16]) (Table 1).

### 2.3. Coprological Methods

The samples were examined using qualitative coprological methods: the Willis flotation technique with a 33% zinc sulfate (ZnSO_4_) solution for detection of lethargic larvae and a Sheather’s sugar solution [17,18,19] for the identification of gastrointestinal parasites and a modified Baermann method for the detection of the L1 larvae of pulmonary nematodes [20]. In addition, a macroscopic examination of all samples was previously performed to assess factors such as consistency, color, existence of blood, mucus and the presence of parasitic forms. The parasites’ identification was based on morphological and morphometric features such as length, width, the posterior and anterior ends of larvae, size, shape, color, shell thickness, surface morphology and content [17,18,19,20,21,22,23,24].

#### Statistical Analysis

The information collected from the questionnaires and the results of the coprological methods carried out were inserted into a file in the Microsoft Excel 2010^®^ program and later imported into the R program, version 3.3.0, with the R Commander extension. Using the R program, the data were analyzed using contingency tables (two-way table) and Pearson’s chi-square test to evaluate the association of the predisposing factors with infections detected in the examined animals. The results were considered statistically significant when the *p*-value was less than 0.05.

Binary multiple univariate generalized linear models (GLMs) were used to test the two islands and species mentioned with the presence of gastrointestinal parasites, lungworms and positivity for zoonotic pathogens (hookworms and *Toxocara* spp.) [25]. The same analysis was performed to test whether the occurrence of infection with certain parasite species was related to the island of origin.

## 3. Results

### 3.1. Sampled Population

On Island A, the study sample comprised 101 canines (67.97% of the total sample on this island) and 49 felines (32.03%). On Island B, the study included 104 canines (60.48%) and 66 felines (39.52%). On Island A, 77% of dogs (80/104) and 71% of cats (35/49) were adults. On Island B, the proportion of adults recorded was 77% in dogs (63/104) and 76% in cats (27/49). In the sample under study, most of the dogs analyzed on Island A were males (60.58%), while for cats, most were females (55.1%). On Island B, 63.37% of the dogs (64/101) were males, and 56.06% of the cats (37/66) were females. In this survey, on Island A, the majority of animals were mixed-breed, with a prevalence of 72.12% in dogs (75/104) and 91.84% in cats (45/49). The same scenario occurred on Island B, where 67.33% of the dogs (68/101) and 86.36% of the cats (57/66) (*p* < 0.05) were mixed-breed (Table 2 and Table 3).

Lifestyle, related to the area of housing/accommodation where the animal lives, was distributed in two classifications: exterior and interior. In addition, another factor considered was the possibility of access to the outside/street for the animals, especially dogs, which can live inside or in yards/gardens but have regular access to the street. On Island A, most dogs lived outside in yards (90%) but did not have access to the streets (93%). In cats, 80% had outdoor housing, in yards or private gardens, with access to the street only allowed for one cat (2%). This was due to the confinement of the majority of cats sheltered in catteries, where this access is denied. On Island B, 83% of dogs (84/101) lived outside, in yards, where outside access was allowed for to 31% (*p* < 0.05). Of the felines, 80% had indoor housing, with outdoor access allowed to 32% (*p* < 0.05) (Table 2 and Table 3).

Regarding cohabitation with other animals in the same space, 94.23% of the dogs (98/104) from Island A cohabited with other animals, while among the cats, the prevalence of cohabitation was 95.92% (47/49) (*p* < 0.05). On Island B, 91.09% of the dogs (92/101) cohabited with other animals, while for the cats, the prevalence of cohabiting animals was 93.94% (62/66) (*p* < 0.05) (Table 2 and Table 3).

Another evaluated criterion was the deworming frequency, with animals distributed across four classifications: dewormed for less than 2 weeks; dewormed for 3–4 weeks (monthly); dewormed for 2–3 months; and dewormed for more than 3 months. This range was based on the totality of responses obtained in the questionnaire, with many of the participants being unaware of the adequate deworming frequency. On Island A, the dogs were frequently dewormed every 2–3 months (50.96%) or every 3–4 weeks (41.35%). For the cats, 57.14% (28/49) were dewormed every 3–4 weeks and 42.86% (21/49) every 2–3 months (*p* < 0.05). On Island B, 59.41% (60/101) of dogs were dewormed in periods of more than 3 months and 22.77% (23/101) every 2–3 months. In cats, 51.52% (34/66) were dewormed in periods of more than 3 months and 36.35% (24/66) every 2–3 months (*p* < 0.05) (Table 2 and Table 3). In sum, the administration of antiparasitic prophylactics, on Island A, was applied mainly monthly or every 2 to 3 months, with protocols established in kennels and catteries and a growing awareness, although still reduced, on the part of tutors. On Island B, deworming was predominantly applied every 2 to 3 months or longer. It is noteworthy that, on this island, tutors with a higher level of education and training were more sensitive to the issue and applied antiparasitic drugs with some regularity, while the rest, who represent the majority, only administrated them annually or had indoor animals for which the probability of occurrence of parasitosis is lower.

Regarding the presence of previously diagnosed diseases, on Island A, 4.81% (5/104) of the canines had concomitant diseases, while in the felines, the prevalence of cats that had concomitant diseases was 2.04% (1/49). On Island B, 7.92% (8/101) of the canines had been previously diagnosed with concomitant diseases, while for the felines, this value was 15.15% (10/66) (Table 2 and Table 3). This diagnosis of concomitant diseases was previously carried out by veterinary clinicians working at clinics, animal shelters or kennels. In general, the sampled animals were considered healthy and without clinical signs that could show disease. Of the few cases in which a concomitant disease was registered, pathologies such as arthrosis were present in older animals (especially dogs), feline asthma and dermatological problems such as folliculitis, yeast infections and allergic dermatitis, which were adequately followed up with and previously treated. For these animals, the obtained results were negative.

On São Miguel Island, 92.31% (96/104) of the dog samples came from kennels and associations, while the remaining 7.69% (8/104) were provided by tutors (*p* < 0.05). In cats, 79.59% (39/49) came from catteries and associations, while the remaining 20.41% (10/49) were provided by tutors (*p* < 0.05). On Terceira Island, 61.38% (62/101) of sampled dogs were accommodated in kennels and associations, while the remaining 38.61% (39/101) had tutors (*p* < 0.05). In cats, 56.06% (37/66) of the samples came from catteries and associations, while the remaining 43.94% (29/66) were provided by tutors (*p* < 0.05) (Table 2 and Table 3).

The presence of parasitic infection was significantly related to the length of time the animals had spent in the kennels/catteries (*p* < 0.05) (Table 4). Both dogs and cats housed in these shelters for more than a year were more likely to develop infection.

### 3.2. Macroscopic Examination

Significantly more infections occurred in animals when parasitic macroscopic forms were observed in the feces (*p* < 0.05). The remaining factors (consistency, color, blood and mucus) had no association with the occurrence of parasite infection (*p* > 0.05) Also, the infected animals usually presented normal feces (Table 5 and Table 6).

### 3.3. Distribution and Prevalence of Gastrointestinal Parasites and Lungworms

Overall, 205 dog samples and 115 cat feces were subjected to microscopic examinations on Islands A and B (Table 3 and Table 4). The overall prevalence of gastrointestinal parasitism in dogs was 53% (108/205), where the results obtained for each parasite were the following: Ancylostomatidae (hookworms) (42.44%), *Trichuris vulpis* (17.56%), *Toxocara canis* (12.68%) and *Cystoisospora* spp. (4.39%) (Figure 2). Of these 108 positive samples, 46 (42.6%) animals were identified with mixed infections, while the remaining 62 (57.4%) were only infected with one parasitic pathogen. The most prevalent parasites were nematodes, followed by protozoa. Island A (São Miguel) showed the highest prevalence of infection with gastrointestinal parasites (65%), and also the highest prevalence of each zoonotic parasite in dogs was attributed to hookworms (51%) and *Toxocara canis* (15.4%) (*p* < 0.05) on Island B (Table 7, Table 8 and Table 9).

**Figure 3:**
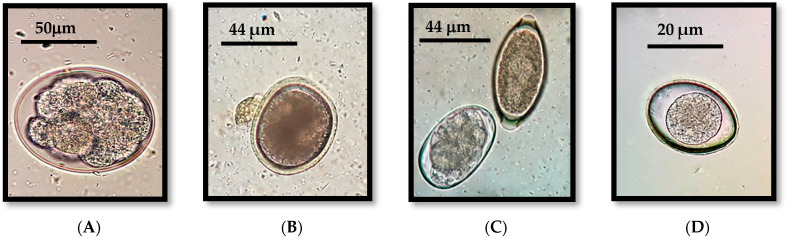
(**A**) Hookworm egg (cat). (**B**) *Toxocara canis* egg (dog). (**C**) Mixed infection with eggs of hookworms (**left**) and *Trichuris vulpis* (**right**) (dog). (**D**) Oocyst of *Cystoisospora* sp. (cat).

**Figure 4:**
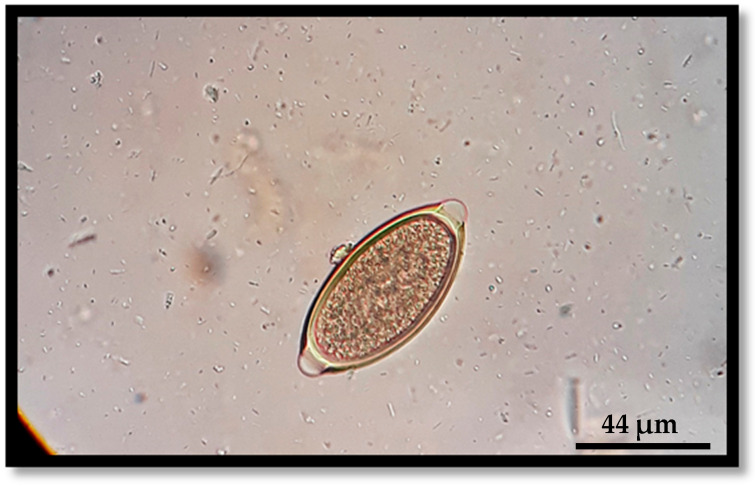
*Trichuris* sp. (cat)—Barrel-shaped and yellow–brown egg (77 × 36 μm) with prominent bipolar end plugs and a smooth shell.

The overall prevalence of gastrointestinal parasitism in cats was also 53% (61/115), and each parasite was registered at the following prevalence: *Toxocara cati* (32.17%), Ancylostomatidae (hookworms) (29.57%), *Cystoisospora* spp. (14.78%) and *Trichuris* sp. (0.87%) (Figure 3 and Figure 4).

Of these 61 positive samples, 26 (42.62%) animals were identified with mixed infections, while the remaining 35 (57.38%) were only infected with one parasitic pathogen. The most prevalent parasites were also nematodes. Island B (Terceira) showed the highest prevalence of infection with gastrointestinal parasites in cats (56%) and zoonotic parasites: *Toxocara cati* (39.4%) and hookworms (38%) (*p* < 0.05) (Table 8, Table 9 and Table 10).

**Figure 5:**
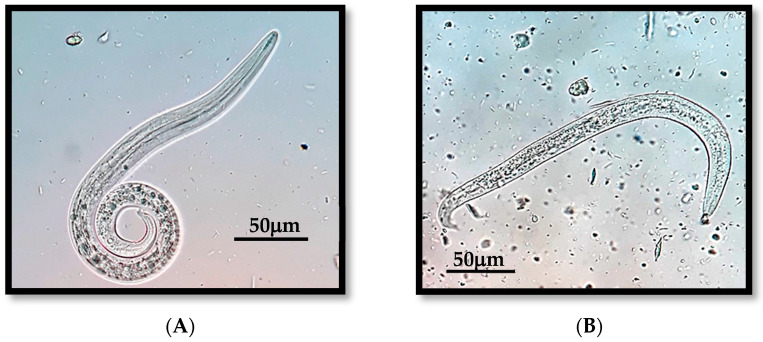
(**A**) First-stage larvae (L1) of *Aelurostrongylus abstrusus* (cat). (**B**) First-stage larvae (L1) of *Angiostrongylus vasorum* (dog).

The prevalence of pulmonary parasitism was 0.49% (1/205) in canines and 20.87% (24/115) in felines, with *Angiostrongylus vasorum* and *Aelurostrongylus abstrusus* being the only species detected in dogs and cats, respectively (Figure 5).

In dogs, only one specimen was identified on Island B (*p* < 0.05). Island B (Terceira) also showed the highest prevalence of infection with lungworms in cats (24.24%) (*p* < 0.05) (Table 7, Table 8, Table 9 and Table 10).

## 4. Discussion

Using the information collected through the questionnaires, it was found that, on both islands, the samples were predominantly made up of adult dogs and cats. This may have been due to the origin of the samples; animals with a more advanced age predominated. Since these animals are more prone to the development of chronic diseases, the low prevalence obtained could lead to the hypothesis that certain diseases are underdiagnosed. Regarding the breed factor, in both species, the samples were mainly from animals of mixed breeds, a finding that is related to the fact that they were mostly rescued from the street. This is a result of the current concern that has developed with regard to relocating and collecting stray or abandoned animals to ensure not only animal welfare but also public health [14]. In the region, dogs and cats frequently cohabit with other animals, usually of the same species, constituting a risk factor for parasitic transmission [2,26,27,28,29,30,31]. The length of time spent in shelters was also related to the presence of parasitic infection since both species housed in these shelters for more than a year are more likely to develop infections. All the animals who entered the shelter were confined in isolation units for 15 days and properly dewormed, suggesting that infection occurs within the facilities through direct contact and the fecal–oral route. This can be explained by the lack of proper prophylactic protocols and the less hygienic conditions that these animals normally live in, combined with the high population density that usually exists in shelters [27,32]. In the future, it would be advisable to carry out parasitological studies of quarantined animals upon their entry into shelters to eliminate the significance of the time spent in the facilities as a risk factor.

Regarding the administration of antiparasitic prophylactics, positive samples were associated with the poor deworming frequency of the affected animals, which constitutes an important risk and prevention factor. Moreover, most of the samples were collected in kennels, catteries and associations, which resulted in a greater number of positive results [25,32,33,34,35]. It is important to highlight the intermittent excretion of parasites, so it would be advisable, in future studies, to analyze three samples from different days in order to obtain a higher prevalence [18,24]. More infections occurred in animals when parasitic forms were observed in feces, which was predictable since the animals were already infected. Moreover, the infected animals usually presented normal feces, which indicates that the presence of infection may not be directly related to macroscopic changes in their feces. It is important to highlight this point since normal feces may hide parasite shedding, and regular fecal monitoring still makes sense as a major tool for the good control of a pet’s gastrointestinal parasites [36].

The present survey detected a high prevalence of gastrointestinal infection in both dogs and cats, probably due to the origin of samples and the ideal climatic conditions for the development of parasites. Similar results were found in other insular territories [2,30,31]. On Greek islands, a study of cats showed endoparasitism’s prevalence at around 58% (Mykonos) and 64% (Skopeles) [25]. On Sardinia Island (Italy), the prevalence of recorded endoparasites was 34.9% (dogs) and 43.4% (cats) [36]. On Mallorca Island (Spain), a study performed on a sample of feral cats recorded a prevalence of 100%, with all animals being parasitized by helminths [37]. On the Philippine islands, the recorded prevalence of intestinal helminths in dogs was 97.45% [38], while the Galápagos islands (which have a similar climate to the Azores) recorded 53.6% of simple infections and 11.4% co-infections [39]. However, it is thought that the lack of awareness among a part of the population regarding the occurrence and risk that these parasites entail for their animals and for public health is the main factor in the manifestation of the results obtained, as verified in other studies carried out in Portugal [2,29,30,31,32]. Furthermore, the environment and population density, together with the sample being mainly composed of stray animals, favor the occurrence of parasites, which justifies the obtained results [14,28,29,30,31].

The higher occurrence of gastrointestinal and zoonotic parasites in dogs from São Miguel (Island A) is most likely due to the large number of stray dogs sampled. However, the opposite scenario occurred on Island B, where a higher prevalence of gastrointestinal and zoonotic parasitism was observed in cats. Although these findings may be attributed to chance, they can also be explained by the hygienic and sanitary conditions in which cattery cats were found. Island B has only one cattery, where the animals are sheltered in indoor facilities with a high and alarming population density, which makes their hygiene difficult and, therefore, promotes the spread of infections. On the other hand, cats from Island A are housed in outdoor catteries with fewer individuals per cage and better hygiene conditions. Furthermore, the island has three centers available to provide shelter to these animals. It could also be possible that differences in veterinary care in terms of prevention, diagnosis and treatment played a certain role, although further investigations are needed to clarify these issues [2,25,32].

Another aspect to consider is the period in which the collection took place. This study was carried out from September to January, and although the Azores archipelago presents high rainfall and relative humidity throughout the year, in September and October, these values usually reach their maximums. It was, in fact, in these months that the number of positive animals detected was higher, with the seasonality factor standing out.

In this survey performed in an insular territory, the high prevalence of hookworm eggs was highlighted due to its potential health risk as a zoonotic disease. In humans, hookworm larvae can penetrate the skin and cause follicular, papular and ephemeral lesions, muscular damage, and eosinophilic enteritis [40]. This can be due to the facilities and origin of the animals, as well as the presence of ideal temperatures and relative humidity, which provide the optimum conditions for their proliferation [41]. The *Toxocara cati* infection in this insular environment is also of importance, given its zoonotic nature, which may lead either to subclinical infections or to different larva migrans syndromes (visceral, ocular and neural), which may have serious clinical manifestations in humans [17,34]. It is also interesting to note that a specimen of *Trichuris* sp. was detected in the cat sample, and this is a rare parasite according to some of the existing literature [16,36]. Another factor that stood out was the important prevalence of *Cystoisospora* spp. in cats of the archipelago, which, in the present survey, was 14.78%. According to ESCCAP (2018), cats that come from catteries or that are in situations with a high population density are at greater risk of contracting protozoan infections [42]. This factor, together with the region’s favorable climatic conditions, may have been the origin of the obtained results.

The prevalence of aelurostrongylosis in the region is also considerable (20.87%), so it should not be neglected and should be included in the list of differential diagnoses of pathologies concerning the respiratory tract in the felids of the Azores archipelago. *A. abstrusus* is a parasite that affects feline lung tissues; severe infections can lead to verminous pneumonia, which can be fatal [43,44,45,46]. This cosmopolitan nematode is the most frequent lungworm diagnosed in felids, being reported in Europe, South America, Australia, the Middle East, Russia, the Far East, the USA, China and Africa [47,48,49,50,51,52,53,54,55,56,57,58,59]. As was said before, studies related to cats in this insular region are practically nonexistent. Therefore, this study contributes to the knowledge of pulmonary nematodes affecting cats in the Azores islands, providing new data on the prevalence and distribution of these parasites. Only one specimen of *A. vasorum* was detected, and studies of this parasite in Portugal are scarce [14,60,61,62,63,64]. Other studies carried out on European islands obtained the following prevalence of *A. vasorum*: 4.6% in Tenerife Island (Spain); 15% in El Hierro Island (Spain) [65]; 18.1% in Aegean islands (Greece); 5.5% in Ionian islands (Greece) [66]; 9.8% in Giglio Island (Italy) [67]; and 3.4% in Sardinia Island (Italy) [68].

This could be due to numerous factors, such as the diagnostic method used. The method of choice used corresponds to the Baermann technique. Despite being a quick and easy-to-perform method, these parasites have peculiarities that often make their detection impossible. Within these particularities, the long pre-patent periods (28 to 108 days) and the intermittent excretion that this parasite can present stand out [18,24]. Therefore, in a future study, it is advisable to collect feces for three successive days, and if possible, repeat the collection after a pre-established period [18,24]. Given these results, it is thought that the use of different diagnostic methods can be a determinant and complementary factor in the detection of these metastrongylids such that, given the high abundance of terrestrial gastropods and paratenic hosts in the region, it is estimated that the prevalence of *A. vasorum* is higher than that determined in this screening [69].

## 5. Conclusions

The present survey was a pioneering one regarding the study of gastrointestinal and pulmonary parasites in populations of domestic carnivores in the Autonomous Region of the Azores. It allowed us to successfully confirm the presence of these helminths and determine associated risk factors, thus remedying the scarcity of studies in this archipelago. Such information is critical, given the potential impact it has on local biodiversity, domestic carnivores and public health due to the identification of zoonotic species while also contributing to new aspects to be explored in works of this nature or others that are intended to pore over the parasitology of companion animals in this insular region.

As future perspectives for research on parasites in domestic carnivores of the Azores, we include (a) sampling the other seven islands to complete the data on the prevalence of gastrointestinal and pulmonary parasites across the Azores archipelago, (b) the evaluation of this prevalence in a sample with more animals from caregivers, (c) the use of or complementing with different diagnosis methods, (d) determining environmental contamination with these zoonotic agents and (e) evaluating the incidence of *Aelurostrongylus abstrusus*, as it is a very reliable indicator of cats’ hunting behavior and the reduction in biodiversity.

## Figures and Tables

**Table 1:** Minimum sample size selection.

Area	Estimated Dog Numbers	Estimated Cat Numbers	Min. Sample Size Dogs/Cats	No. of Collected Samples from Dogs	No. of Collected Samples from Cats
São Miguel	- *	- *	14/12	104	49
Terceira	- *	- *	14/12	101	66
				205	115

* No official records at the moment.

**Table 2:** Physical and parasitological features of the sampled population on Island A. Significant factors are highlighted with *p*-values in bold.

	Dogs	*p*-Value	Cats	*p*-Value
	*n* (%)		*n* (%)	
Age				
Young	24 23	*p* = 0.08	14 29	*p* = 0.062
Adult	80 77		35 71	
Sex				
Female	41 39.42	*p* = 0.17	27 55.1	*p* = 0.21
Male	63 60.58		22 44.9	
Breed				
Undeterminate	75 72.12	*p* = 0.06	57 91.84	*p* = 0.4
Purebreed	29 27.88		9 8.16	
Lifestyle				
Exterior	94 90	*p* = 0.11	39 80	*p* = 0.34
Interior	10 10		10 20	
Exterior Access				
Yes	7 7	***p* = 1.88 × 10^−6^**	1 2	***p* = 0.028**
No	97 93		48 98	
Cohabitation				
Yes	98 94.23	***p* = 0.038**	47 95.92	***p* = 0.02**
No	6 5.77		2 4.08	
Deworming Frequency				
<2 weeks	5 4.82	***p* = 0.033**	0 0	***p* = 0.015**
3–4 weeks	43 41.35		28 57.14	
2–3 months	53 50.96		21 42.86	
>3months	3 2.88		0 0	
Diagnosed Diseases				
Yes	5 4.81	*p* = 0.23	1 2.04	*p* = 0.09
No	99 95.19		48 97.96	
Origin of Samples				
Kennels/Catteries/Associations	96 92.31	***p* = 0.03**	39 79.59	***p* = 1.93 × 10^−6^**
Tutors	8 7.69		10 20.41	
**Total**	**104** **100**		**49** **100**	

**Table 3:** Physical and parasitological features of the sampled population on Island B. Significant factors are highlighted with *p*-values in bold.

	Dogs	*p*-Value	Cats	*p*-Value
	*n* (%)		*n* (%)	
Age				
Young	23 23	*p* = 0.1	16 24	*p* = 0.068
Adult	78 77		50 76	
Sex				
Female	37 36.63	*p* = 0.07	37 56.06	*p* = 0.2
Male	64 60.58		29 43.94	
Breed				
Undeterminate	68 67.33	***p* = 1.04 × 10^−4^**	57 86.36	***p* = 0.04**
Purebreed	33 32.67		9 13.64	
Lifestyle				
Exterior	84 83	*p* = 0.6	13 20	*p* = 0.52
Interior	17 17		53 80	
Exterior Access				
Yes	31 31	***p* = 0.01**	21 32	***p* = 0.038**
No	70 69		45 68	
Cohabitation				
Yes	92 91.09	***p* = 0.018**	62 93.94	***p* = 0.03**
No	9 8.91		4 6.06	
Deworming Frequency				
<2 weeks	3 2.97	***p* = 1 × 10^−6^**	3 4.55	***p* = 2.3 × 10^−10^**
3–4 weeks	15 14.85		5 7.58	
2–3 months	23 22.77		24 36.35	
>3 months	60 59.41		34 51.52	
Diagnosed Diseases				
Yes	8 7.92	*p* = 0.31	10 15.15	*p* = 0.19
No	93 92.08		56 84.85	
Origin of Samples				
Kennels/Catteries/Associations	62 61.38	***p* = 4.04 × 10^−15^**	37 56.06	*p* = 1.21 × 10^−5^
Tutors	39 38.61		29 43.94	
**Total**	**101** **100**		**66** **100**	

**Table 4:** Length of time spent by animals in kennels and catteries. Significant factors are highlighted with *p*-values in bold.

	Dogs	*p*-Value	Cats	*p*-Value
	*n* (%)		*n* (%)	
Length of time spent in kennels/catteries				
Island A				
<1 month	9 9.4	***p* = 0.007**	11 28.2	***p* = 0.012**
2–12 months	5 5.2		3 7.7	
1–5 years	75 78.1		22 56.4	
>5 years	7 7.3		3 7.7	
Total	96 100		39 100	
Island B				
<1 month	5 8.1	***p* = 0.000**	8 21.6	***p* = 0.020**
2–12 months	9 14.5		5 13.5	
1–5 years	39 62.9		22 59.5	
>5 years	9 14.5		2 5.4	
**Total**	**62** **100**		**37** **100**	

**Table 5:** Macroscopic features of sampled feces from Island A. Significant factors are highlighted with *p*-values in bold.

	Dogs	*p*-Value	Cats	*p*-Value
	*n* (%)		*n* (%)	
Island A				
Consistency				
Liquid	2 1.9	*p* = 0.94	2 4.1	*p* = 0.92
Very Soft	3 2.9		4 8.2	
Soft	17 16.4		8 16.3	
Formed	82 78.8		35 71.4	
Color				
Normal	102 98.1	*p* = 0.72	48 98	*p* = 0.55
Abnormal	2 1.9		1 2	
Blood				
Presence	4 4	*p* = 0.29	3 6.1	*p* = 0.2
Absence	100 96		46 93.9	
Mucus				
Presence	3 2.9	*p* = 0.54	2 4	*p* = 0.32
Absence	101 97.1		47 96	
Parasitic Forms				
Presence	7 6.7	***p* = 0.003**	4 8.2	***p* = 0.04**
Absence	97 93.3		45 91.8	
Total	104 100		49 100	
Infected animals				
	**Dogs**		**Cats**	
	***n*** **(%)**		***n*** **(%)**	
Feces				
Normal	51 75		19 79	
Abnormal	17 25		5 21	
**Total**	68 100		24 100	

**Table 6:** Macroscopic features of sampled feces from Island B. Significant factors are highlighted with *p*-values in bold.

	Dogs	*p*-Value	Cats	*p*-Value
	*n* (%)		*n* (%)	
Island B				
Consistency				
Liquid	4 4	*p* = 0.83	4 6	*p* = 0.79
Very Soft	4 4		5 7.6	
Soft	14 13.9		11 16.7	
Formed	79 78.1		46 69.7	
Color				
Normal	98 97	*p* = 0.49	64 97	*p* = 0.73
Abnormal	3 3		2 3	
Blood				
Presence	5 5	*p* = 0.55	4 6	*p* = 0.38
Absence	96 95		62 94	
Mucus				
Presence	2 2	*p* = 0.75	2 3	*p* = 0.51
Absence	99 98		64 97	
Parasitic Forms				
Presence	4 4	***p* = 0.04**	6 9	***p* = 0.03**
Absence	97 96		60 91	
Total	101 100		66 100	
Infected animals				
	**Dogs**		**Cats**	
	***n*** **(%)**		***n*** **(%)**	
Feces				
Normal	33 83		29 78	
Abnormal	7 17		8 22	
**Total**	40 100		37 100	

**Table 7:** Microscopic fecal examination: number (n) and percentage (%) of positive dogs for different parasites on Islands A (São Miguel) and B (Terceira).

Parasite	Island A (*n* = 104) *n*/%	Island B (*n* = 101) *n*/%	Total (*n* = 205) *n*/%
Ancylostomatidae (hookworms)	53 (51)	34 (33.7)	87 (42.4)
*Trichuris vulpis*	20 (19.2)	16 (15.8)	36 (17.6)
*Toxocara canis*	16 (15.4)	10 (9.9)	26 (12.7)
*Cystoisospora* spp.	7 (6.7)	2 (1.98)	9 (4.4)
*Angiostrongylus vasorum*	0 (0)	1 (1)	1(0.5)
Mono-infections	44 (64.7)	18 (45)	62 (57.4)
Mixed infections	24 (35.3)	22 (55)	46 (42.6)
Total number of positive dogs	68 (65)	40 (40)	108 (53)

**Table 8:** A statistical analysis evaluating two factors (the island where the animals lived and the species) in relation to the different infections detected in the study. Significant factors are highlighted with *p*-values in bold.

	Positive for Gastrointestinal Parasites	Positive for Lungworms	Zoonotic Infections
Variable	***n* (Total)**	***n* (Total)**	***n* (Total)**
	**%**	**%**	**%**
	***GLM p-value***	***GLM p-value***	***GLM p-value***
*Species*			
Dogs vs. Cats	169	25	152
	53	8	47.5
	***0.025***	***0.000***	*0.310*
*Island* *—* *Dogs*			
São Miguel vs. Terceira	108	1	100
	53	0.49	49
	***0.009***	***0.028***	***0.001***
*Island* *—* *Cats*			
São Miguel vs. Terceira	61	24	52
	53	21	45
	***0.012***	*0.213*	*0.083*

**Table 9:** A statistical analysis evaluating the island of origin concerning the occurrence of infection with certain parasite species. Significant factors are highlighted with *p*-values in bold.

	Positive
Variable	***n* (Total)**
	%
	***GLM p-value***
Hookworms—Island (Dogs)	
São Miguel vs. Terceira	87
	42
	***0.031***
Hookworms—Island (Cats)	
São Miguel vs. Terceira	34
	30
	***0.007***
*Toxocara canis*—Island (Dogs)	
São Miguel vs. Terceira	26
	13
	*0.166*
*Toxocara cati*—Island (Cats)	
São Miguel vs. Terceira	37
	32
	*0.058*
*Trichuris vulpis*—Island (Dogs)	
São Miguel vs. Terceira	36
	18
	*0.325*
*Trichuris* sp.—Island (Cats)	
São Miguel vs. Terceira	1
	0.9
	*0.574*
*Cystoisospora* spp.—Island (Dogs)	
São Miguel vs. Terceira	9
	4.4
	*0.064*
*Cystoisospora* spp.—Island (Cats)	
São Miguel vs. Terceira	17
	15
	*0.178*
*Aelurostrongylus abtrusus*—Island (Cats)	
São Miguel vs. Terceira	24
	21
	*0.213*
*Angiostrongylus vasorum*—Island (Dogs)	
São Miguel vs. Terceira	1
	0.5
	***0.028***

**Table 10:** Microscopic fecal examination: number (n) and percentage (%) of positive cats for different parasites on Islands A (São Miguel) and B (Terceira).

Parasite	Island A (*n* = 49) *n*/%	Island B (*n* = 66) *n*/%	Total (*n* = 115) *n*/%
*Toxocara cati*	11 (22.5)	26 (39.4)	37 (32.2)
Ancylostomatidae	9 (18.4)	25 (37.9)	34 (29.6)
*Cystoisospora* spp.	5 (10.2)	12 (18.2)	17 (14.8)
*Trichuris* sp.	0 (0)	1 (1.5)	1 (0.9)
*Aelurostrongylus abtrusus*	8 (16.3)	16 (24.2)	24 (20.9)
Mono infections	18 (75)	17 (46)	35 (57.4)
Mixed infections	6 (25)	20 (54)	26 (42.6)
Total number of positive cats	24 (49)	37 (56)	61 (53)

## Data Availability

Raw data supporting the conclusions of this study are available from the authors upon request.
